# Ghrelin levels in dairy calves: Influence of postnatal separation from the dam in commercial farms

**DOI:** 10.3168/jdsc.2026-1029

**Published:** 2026-07-06

**Authors:** Cynthia López-Novo, Silvia Rojo-Montejo, Nicolás Mejuto-Vázquez, Margarita Rico, Ruth Rodríguez-Bermúdez, Francisco Javier Diéguez, Ramiro Fouz

**Affiliations:** Department of Anatomy, Animal Production and Veterinary Clinical Sciences, Faculty of Veterinary Science, University of Santiago de Compostela, Lugo, 27002, Spain

## Abstract

Conventional management systems in dairy farms are based on the separation of the dam from the calf immediately after calving. However, early contact with the dam may ease the nutritional transition by allowing more frequent colostrum and milk intake, potentially influencing regulation of ghrelin, a hormone whose secretion increases during fasting, serving as a potential biomarker of hunger. This longitudinal study aimed to evaluate serum ghrelin concentrations in dairy calves managed under 2 different systems: (1) conventional separation from the dam immediately after birth and (2) dam–calf contact for at least 24 h postpartum. Data were collected from 8 Holstein dairy farms in Galicia, Spain. Serum samples were taken from 24 calves in the dam–calf contact system at 3 time points: just before separation (T1), 2 d after separation (T2), and 7 d after separation (T3). Additionally, 24 age-matched calves in the conventional system were sampled at the same postpartum time points. Ghrelin concentrations were measured using a commercial ELISA kit, and the statistical analysis was conducted using a linear mixed model. Results revealed that calves allowed dam contact exhibited significantly lower ghrelin concentrations at all sampling times compared with those separated immediately after birth. These findings suggest that early maternal contact may reduce hunger-driven ghrelin secretion, likely due to improved satiety. This study highlights the influence of early-life management on endocrine responses in dairy calves and supports the potential benefits of dam–calf contact systems. Further research is needed to explore the long-term effects on calf development and production efficiency.

## Graphical Abstract

**Figure:**
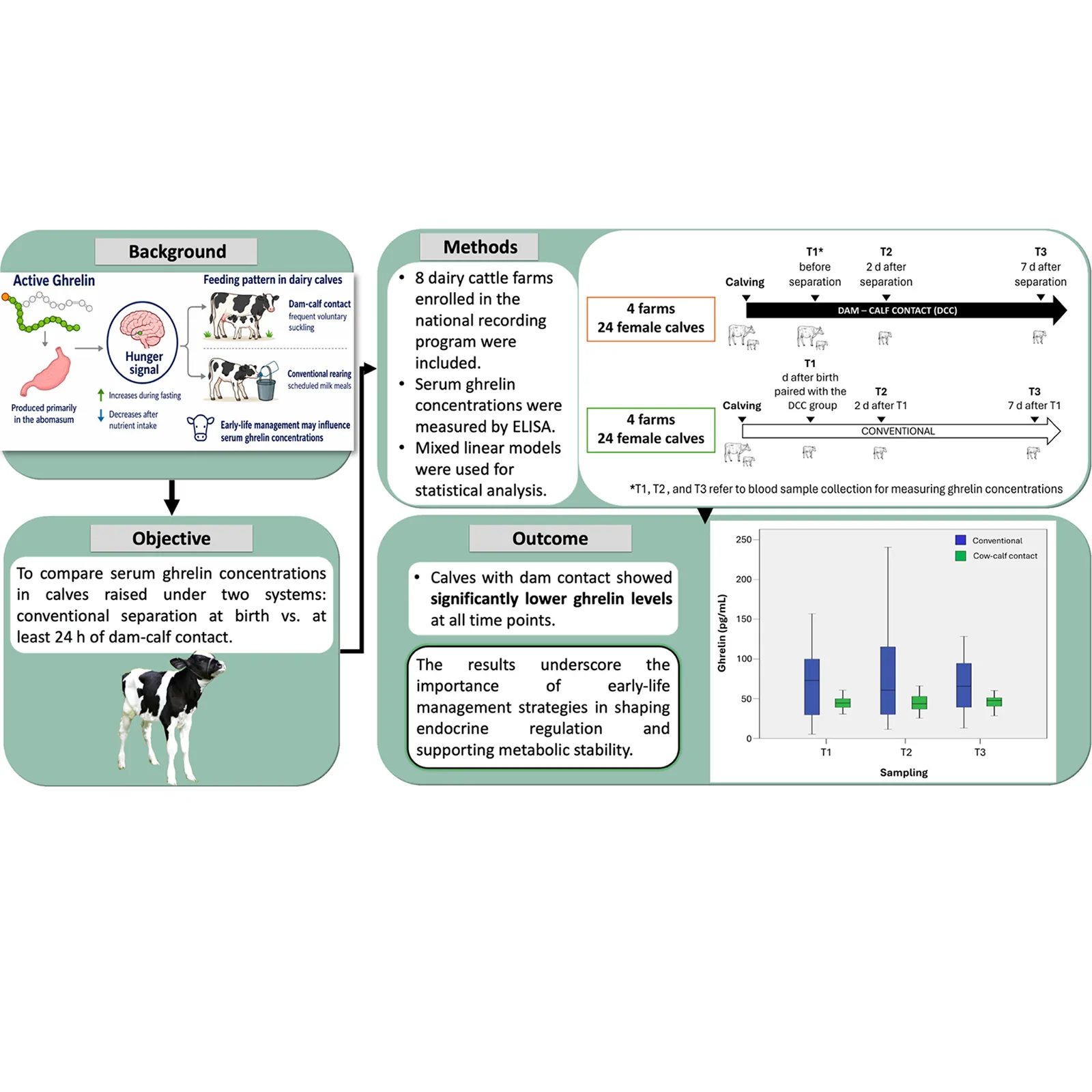

Ghrelin is increasingly recognized as a key hormone in mammals, playing a crucial role in regulating appetite, energy balance, and metabolism (Hayashida et al., 2001). Its secretion rises during fasting, stimulating feed intake when access to food is restored (Wertz-Lutz et al., 2006). Notably, ghrelin concentrations are negatively associated with ad libitum feed intake (Bohan et al., 2007). In ruminants, ghrelin is primarily produced in the abomasum but is also synthesized in other tissues, including the intestines, pancreas, and hypothalamus (Hayashi et al., 2020).

In dairy calves, ghrelin is particularly important during early life, as calves must transition from in-utero nutrient supply to independent feeding. During this phase, ghrelin not only stimulates feed intake but also supports growth hormone release, contributing to growth and metabolic adaptation. Furthermore, ghrelin is involved in glucose homeostasis and lipid metabolism (Sugino et al., 2026). Ghrelin concentrations in dairy calves are possibly higher than in older cattle, because calves have a lower gastric capacity and, thus, consume a lower amount of feed that requires a higher frequency of secretion (Heredia et al., 2015). Nevertheless, this may not always be the case, as Miura et al. (2004) reported lower ghrelin concentrations in 3-mo-old Holstein calves than in adult cattle. Therefore, the ghrelin secretory system may be immature in such young calves and lead to varying results. Previous studies on conventionally reared suckling Holstein calves report ghrelin concentrations of 60 pg/mL in blood serum (Heredia et al., 2015) and 70 pg/mL in plasma (Itoh et al., 2005). Nevertheless, to the best of our knowledge, no studies have investigated the daily variation in ghrelin concentrations in suckling dairy calves in relation to feeding. However, studies in sheep and humans have identified a ghrelin surge that occurs immediately before feeding (Sugino et al., 2002; Broglio and Ghigo, 2006). After meals, the concentrations of this hormone have been reported to decrease in cows 1 h after feed intake, followed by a return to pre-feeding values (Hayashida et al., 2001). Similarly, Miura et al. (2004) observed a decrease in ghrelin concentrations after meals in cows, but not in 3-mo-old dairy calves. Thus, variations in ghrelin concentrations in calves might not follow the same pattern observed in adult animals.

Management practices immediately after birth can influence both the metabolic and endocrine status of dairy calves. Conventional systems often separate calves from their dams shortly after calving, whereas alternative systems allow for dam–calf contact for a variable period postpartum (Beaver et al., 2019). Early contact with the dam, which enables more frequent colostrum and milk intake during the first few days of life (Miller-Cushon and DeVries, 2015), may confer a nutritional advantage to calves. Artificially reared calves usually receive milk replacer only twice a day, a schedule that may leave some individuals insufficiently nourished or satiated. This could result in higher ghrelin concentrations, as reported in dairy calves and sheep subjected to restricted intake compared with groups fed ad libitum or with high planes of nutrition (Sugino et al., 2002; Bohan et al., 2007). Furthermore, early maternal presence could support the development of feeding behavior (Veissier et al., 2013), which might also have implications for ghrelin secretion.

The latest European Food Safety Authority statement on calf welfare recommends allowing calves to remain with the dam for at least 1 d postpartum, while encouraging the progressive extension of this contact period given the various welfare and developmental benefits with which this practice has been associated. However, it also highlights the need for further research to better understand its practical implications (Nielsen et al., 2023). Although some research has highlighted the broader positive effects of early-life dam–calf contact on calf development and welfare (Meagher et al., 2019), its specific effects on ghrelin concentrations remain poorly understood. In this context, ghrelin represents a particularly suitable biomarker, as its secretion is closely linked to feeding patterns and nutrient availability.

Accordingly, this study aims to assess serum ghrelin concentrations in dairy calves under 2 different management systems: (1) calves separated from their mothers immediately after birth (conventional; **CONV**) and (2) calves that remained with their mothers for at least 24 h postpartum (dam–calf contact; **DCC**); given that, as aforementioned, the main differences between these systems are directly related to milk access and feeding dynamics. Conventionally reared calves are typically fed large volumes of milk replacer at scheduled intervals followed by extended fasting periods between meals. In contrast, calves maintained with their dams can suckle voluntarily, leading to a more fractionated and frequent feeding pattern (Miller-Cushon and DeVries, 2015). Provided that circulating ghrelin increases during fasting and decreases following nutrient intake, we hypothesized that calves managed under a dam–calf contact system would exhibit consistently lower serum ghrelin concentrations than calves subjected to conventional early separation.

The study was carried out in Galicia (northwestern Spain) during 2022 and 2023. The study used a targeted convenience sampling approach to select dairy farms from those enrolled in the Dairy Herd Improvement Program (**DHIP**), all exclusively housing Holstein cattle. Given the low prevalence of farms adopting the DCC system in the region (1.2%), only 4 available met the strict inclusion criteria for our study, which were that farms should (1) allow DCC for a minimum of 24 h postpartum; (2) maintain comprehensive, consistent, and verifiable records related to animal health, reproduction, and production; and (3) follow standardized management protocols that ensure the integrity of the dam–calf bond during the study period. Regarding the first criterion, the mean age of calves at separation in the 4 DCC farms was 3.65 d (SD = 3.17; median = 3.00; interquartile range = 3.00).

These 4 farms implementing the DCC system were matched with 4 conventional farms, which perform immediate separation of the cow and calf following parturition. The matching process ensured comparability between the 2 groups based on key farm characteristics, including herd size, production volume, reproductive management, and available health and performance data. This approach was designed to minimize potential confounders, ensuring valid comparisons between the management systems.

The mean herd size (cows ≥2 yr of age) in the studied farms was 162 (range: 38–398). Apart from the differences in early-life calf management, all participating farms operated under comparable management conditions. Calving protocols were broadly standardized: pregnant cows were moved to individual maternity pens ∼24 h before the expected calving date, where continuous monitoring was ensured. On farms allowing DCC, calves remained continuously in full contact with their dams, except during milking, and had unrestricted access to suckling until separation. After dam–calf separation, calves from all farms were kept in indoor individual calf pens, where they had unlimited access to water and starter feed and were fed milk replacer twice daily.

On farms allowing DCC, serum samples were taken from 24 Holstein female calves at 3 time points: just before separating them from the dam (on average, 3.65 d postpartum; **T1**), 2 d after separation (**T2**), and 7 d after separation (**T3**). On CONV farms, samples were taken from another 24 Holstein female calves at the same time points, with each sample paired to one from a calf of the same age from the DCC group. The number of calves sampled per farm varied according to the availability of eligible animals within each herd at the time of sampling and differences in herd size among matched farms. Specifically, CONV farms contributed 5, 10, 6, and 3 calves, whereas DCC farms contributed 5, 11, 6, and 2 calves. Blood samples were collected by jugular venipuncture into anticoagulant-free evacuated tubes and centrifuged at 224 × *g* for 15 min at 4°C. Sera were frozen at −20°C until tested by ELISA. Blood samples were taken ∼1 h after the morning meal, which took place every day between 0800 h and 1000 h, so that an interference in the working routine of the farms was not caused.

The study exclusively included calves from clinically healthy dams. Calf inclusion was contingent upon both the mother and the neonate remaining free from visible signs of disease. The sampling was performed by veterinary researchers, who performed a complete anamnesis of the mother and its calf before sampling. Moreover, maternal health status was assessed through DHIP records, farmer surveys, and treatment records; any indication of a health issue in the dam resulted in the immediate exclusion of the corresponding calf from the study. Consequently, 13 calves (7 from CONV farms and 6 from DCC) were excluded due to occurrence of calf diarrhea or dam mastitis. In such cases, excluded dyads were replaced by the next available healthy calf from the same farm.

For the sampled calves, data on colostrum and milk intake were recorded. Thus, colostrum quality was analyzed using a refractometer and only colostrum with a Brix value over 20 degrees was administered to the calves. On the 4 CONV farms, the calves were fed at least 4 L of colostrum within the first 6 h of life. On DCC farms, colostrum intake was monitored by the farmer through direct observation, assessing the time of first access to the udder, the number of suckling events after birth, and the calf's suckling behavior, as well as a general assessment of calf vigor. Regarding milk intake, the administration of milk replacer after separation was common to all farms, fed via nipple at a rate of 2 daily feedings (12 h apart) of 3 L each, with a concentration of 125 g of commercial milk powder (20%–24% crude protein and 18%–22.5% crude fat).

Furthermore, for each calf, information on the dam's parity and calving difficulty was gathered from the DHIP. Calving number was divided into 3 categories (1, 2, ≥3). Calving difficulty, according to the DHIP, was classified into 5 categories: (1) easy birth without assistance, (2) normal—slight traction, (3) difficult—strong traction, (4) cesarean section or fetotomy with normal presentation, and (5) abnormal calf presentation. However, in this study, only type 1 (easy) and 2 (normal) calvings were observed.

Serum ghrelin concentrations were determined using a commercial competitive bovine ELISA kit (Cloud-Clone Corp., Houston, TX). All procedures were performed in strict accordance with the protocol provided with the kit, including reagent preparation, incubation conditions, and reading parameters. To ensure analytical consistency, all samples were processed under standardized laboratory conditions and by the same laboratory technician, minimizing interoperator variability. Calibration curves were generated using the standards supplied by the manufacturer, and optical density was measured at 450 nm using a microplate reader. Ghrelin concentrations were calculated using 4-parameter logistic regression based on the calibration curves.

Ghrelin concentrations were analyzed using a linear mixed-effects model (MIXED procedure, SPSS 27, SPSS Inc., Chicago, IL). Farm type (CONV vs. DCC), sampling time (T1, T2, T3), and their interaction were included as fixed effects. The significance of fixed effects was assessed using Type III tests; the threshold of significance was *P* < 0.05. Estimated marginal means were obtained from the model to describe group differences. The initial model also included calving difficulty (score 1 vs. 2), dam parity (1, 2, ≥3), and calf age at the first sampling as covariates; however, these variables were not retained in the final model, as they were not significant (*P* > 0.05).

Repeated measurements were specified at the calf level, with calf identity nested within farm as the subject term. Farm (herd) was included as a random effect to account for potential clustering of observations within herds, and a compound symmetry covariance structure was assumed to account for within-animal correlations over time.

Serum ghrelin concentrations recorded in the 2 types of farms throughout the 3 sampling periods are shown in Figure 1. The linear mixed-effects model revealed a significant main effect of farm type on ghrelin concentrations (*F*_1,52.53_ = 12.60; *P* = 0.001; Table 1), indicating that the management system exerted a consistent influence across the study period. Calves in the DCC system exhibited significantly lower ghrelin concentrations compared with those in the CONV system (Table 2). The magnitude of this effect, as estimated from the linear mixed-effects model, was reflected in a reduction of −59.40 pg/mL (95% CI: −100.89 to −17.91; SE = 20.96; *P* = 0.005) in calves from DCC farms compared with those from CONV farms.

**Figure 1:**
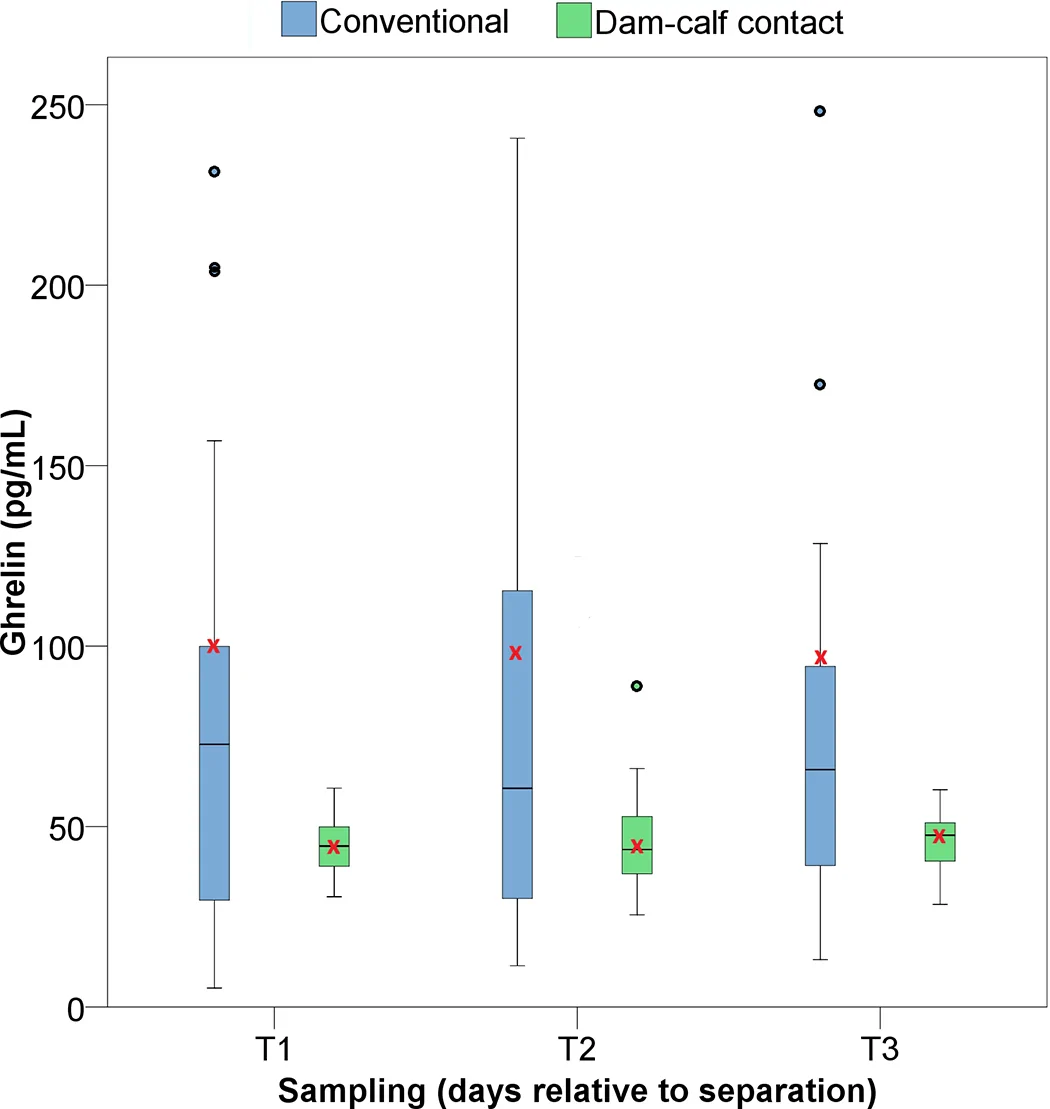
Descriptive boxplots of serum ghrelin concentrations (pg/mL) in neonatal dairy calves from conventional (CONV) and dam–calf contact (DCC) systems across sampling times (n = 4 farms per system; 24 calves per farm type at each time point). The central line within each box represents the median, and the box limits indicate the interquartile range. Whiskers extend to the minimum and maximum values, excluding outliers, represented by dots. Mean values are indicated by a red “X.” Sampling times were defined as T1, immediately before separation (mean 3.65 d postpartum); T2, 2 d after separation; and T3, 7 d after separation. To improve visual readability, 2 extreme observations from the CONV group at T2, with ghrelin concentrations above 250 pg/mL, were omitted from the graphical display. However, these values were retained in the data set and included in all statistical analyses.

**Table 1:** Results of the linear mixed-effects model evaluating the effects of farm type and sampling time on serum ghrelin concentrations in neonatal dairy calves^1^

Effect	Numerator df	Denominator df	*F*	*P*-value
Intercept	1	52.53	87.56	<0.001
Farm type (DCC vs. CONV)	1	52.53	12.60	0.001
Sampling time (T1–T3)	2	102.75	0.03	0.975
Farm type × time	2	102.75	0.05	0.948

1 Sampling times in dam–calf contact (DCC) farms were defined as follows: T1 = immediately before dam–calf separation (mean 3.65 d postpartum); T2 = 2 d after separation; and T3 = 7 d after separation. In conventional (CONV) farms, calves were age-matched to those from DCC farms at each sampling point. A total of 4 farms per system were included, with 24 calves per farm type sampled at each time point.

**Table 2:** Estimated marginal means (±SE) and 95% CI for serum ghrelin concentrations in neonatal dairy calves from conventional (CONV) and dam–calf contact (DCC) systems, derived from a linear mixed-effects model including farm type, sampling time, and their interaction^1^

Farm type	Estimated mean (pg/mL)	SE	95% CI
CONV	101.06	11.01	78.96–123.16
DCC	45.48	11.14	23.15–67.81

1 Sampling times were defined as follows: T1 = immediately before dam–calf separation (mean 3.65 d postpartum); T2 = 2 d after separation; and T3 = 7 d after separation. A total of 4 farms per system were included, with 24 calves per farm type sampled at each time point.

The absence of a significant farm type × sampling time interaction (F_2,102.75_ = 0.05; *P* = 0.948) indicates that this difference between management systems remained consistent across all 3 sampling points. No significant main effect of sampling time was observed (F_2,102.75_ = 0.03; *P* = 0.975; Table 1).

Overall, the findings of this study reveal that dairy calves raised under DCC management systems consistently exhibit lower serum ghrelin concentrations than calves managed under CONV conditions. This difference remained evident across all sampling times, including T2 and T3, suggesting that even brief early maternal contact exerts a sustained influence on ghrelin secretion during the neonatal period. Moreover, ghrelin concentrations observed in DCC calves were lower not only compared with those of CONV calves in the present study, but also compared with values previously reported for suckling Holstein calves in the literature (60–70 pg/mL; Itoh et al., 2005; Heredia et al., 2015). These reduced ghrelin concentrations in DCC calves may be explained by multiple interacting factors. One of the primary explanations is the unrestricted access to colostrum and milk provided by dam contact, which likely enhances satiety and reduces the physiological need for ghrelin-driven hunger signaling. In this regard, ghrelin concentrations are known to rise during fasting and decrease following feed intake (Wertz-Lutz et al., 2006). Similarly, higher ghrelin concentrations have been observed in dairy calves and sheep maintained on lower nutritional planes or restricted intake (Sugino et al., 2002; Bohan et al., 2007). Therefore, the increased milk intake facilitated by the maternal presence likely suppresses ghrelin secretion in these calves throughout the 3 time points included in the study, in contrast with calves separated immediately after birth, for whom milk was always supplied across 2 daily meals. Moreover, an additional noteworthy finding was the markedly lower variability in ghrelin concentrations observed in calves from DCC farms compared with CONV calves. A plausible explanation for these results is the buffering capacity of colostrum (McGrath et al., 2016). In this regard, calves that remain with their mothers, compared with those separated after birth, consume a greater amount of colostrum and transitional milk. Presence of ghrelin in colostrum and milk in measurable amounts suggests that it is a critical compound for the metabolic activity of newborn calves and functions transiently to regulate the activity of some physiological processes until the endocrine system of the new calves starts to function independently (Alhojaily, 2014). Consequently, although a direct relationship between milk-derived ghrelin and circulating concentrations has not been established, the intake of maternal milk might promote more stable circulating ghrelin concentrations in suckling calves compared with those artificially reared from birth. Therefore, early maternal contact might act as a physiological buffer by providing a more continuous feeding pattern during the first days of life. Moreover, unlike the periodic hunger–satiety cycles associated with restricted artificial feeding, the more frequent and self-regulated intake in the DCC group may promote a more stable metabolic state. All this could be reflected in the lower inter-individual variability observed in ghrelin concentrations. The persistence of this reduced variability after separation further indicates that early differences in feeding patterns may have short-term effects on endocrine regulation during the neonatal period.

On another note, natural suckling may promote the establishment of a more diverse and robust gut microbiota compared with calves separated immediately after birth (Diddeniya et al., 2024). In fact, several mechanisms through which microbiota may influence ghrelinergic signaling have been proposed, and both positive and negative associations have been reported between the presence of specific bacterial genera in the gut and circulating ghrelin concentrations (Schalla and Stengel, 2020). Thus, contact with the dam, which allows the calf to receive maternal microbial inoculation, fostering gut development and supporting more stable hormonal regulation, could contribute to the consistently lower ghrelin concentrations observed in DCC calves.

The results of this study align with previous research indicating that social contact and consumption of whole milk during the neonatal period, both characteristics of DCC systems, can positively influence feeding behavior, metabolic programming, and overall development (Van Amburgh et al., 2008; Okkema, 2024). As previously mentioned, few studies have assessed ghrelin concentrations in dairy calves. Notably, Bohan et al. (2007) suggested that reduced feed intake elevates ghrelin concentrations, potentially stimulating appetite to encourage feed consumption. Moreover, ghrelin concentrations are negatively associated with nonesterified fatty acids (**NEFA**; Wertz-Lutz et al., 2006), which have been linked to increased energy intake (van Zyl et al., 2025). Consistently, van Zyl et al. (2025) and McPherson et al. (2025) reported higher NEFA concentrations in DCC calves than in CONV ones, in line with the lower ghrelin concentrations observed in DCC calves in our study. Thus, these results suggest that unrestricted early access to milk may improve energy utilization efficiency, thereby reducing the reliance on ghrelin to regulate feed intake. Additionally, ghrelin concentrations remained stable across sampling times (T1–T3) in both management systems, suggesting that ghrelin regulation is established early in the neonatal period and maintained regardless of separation timing.

The implications of these findings are substantial for both calf welfare and production efficiency. Lower ghrelin concentrations in DCC calves suggest that these animals may experience reduced hunger due to increased milk consumption and, thus, lower hunger-driven stress and better satiety, potentially leading to improved welfare outcomes. Moreover, the potential higher milk consumption in DCC calves may lead to improved gut development, which could also contribute to better immune function and disease resistance in early life (Hammon et al., 2020). From a production standpoint, calves experiencing early maternal contact and exhibiting more stable metabolic regulation may demonstrate improved growth performance (van Zyl et al., 2025), which may lead to improved future milk yields (Van De Stroet et al., 2016).

Although this study provides valuable insights into the relationship between postnatal management and ghrelin regulation, some limitations should be noted. The sample size was relatively modest, and the study was geographically restricted to a single region. Thus, the analysis of ghrelin concentrations in calves should be extended to a broader range of production systems to confirm the generalizability of our findings. Moreover, despite the significant results, the lack of a baseline ghrelin measurement immediately postpartum (before colostrum intake or dam interaction) represents a methodological constraint. However, it must be considered that our first sampling (T1) already revealed a marked suppression of ghrelin in the DCC group, a difference that remained remarkably stable through T2 and T3, which is highly suggestive of an early organizational effect of the management system rather than stochastic baseline variations. Nevertheless, future research should focus on establishing baseline ghrelin values during this critical early period. Additionally, exploring other metabolic and stress-related biomarkers, in conjunction with ghrelin, could offer a more comprehensive understanding of the physiological adjustments in response to early-life management practices.

In conclusion, this study demonstrates that DCC significantly influences ghrelin regulation in dairy calves, with calves exposed to maternal contact consistently showing lower ghrelin concentrations. These findings support an association between early feeding patterns, milk access, and metabolic signaling, suggesting that neonatal management conditions influence endocrine regulation and physiological adaptation during early life. Further research is needed to clarify the long-term effects of DCC on calf development and production efficiency.
